# ORAL CONCURRENT SESSION 3: Ultrasound and Genetics

**DOI:** 10.1002/pmf2.12007

**Published:** 2025-01-05

Abstracts 29 – 38

THURSDAY

January 30, 2025

1:30 PM – 4:00 PM

Aurora Ballroom CD

MODERATORS

Shani Delaney, MD

Angie C. Jelin, MD

